# Development of a Generic PBK Model for Human Biomonitoring with an Application to Deoxynivalenol

**DOI:** 10.3390/toxins15090569

**Published:** 2023-09-13

**Authors:** Sylvia Notenboom, Rudolf T. Hoogenveen, Marco J. Zeilmaker, Annick D. Van den Brand, Ricardo Assunção, Marcel J. B. Mengelers

**Affiliations:** 1 National Institute for Public Health and the Environment (RIVM), 3721 BA Bilthoven, The Netherlands; rudolf.hoogenveen@rivm.nl (R.T.H.); marco.zeilmaker@rivm.nl (M.J.Z.); annick.van.den.brand@rivm.nl (A.D.V.d.B.); marcel.mengelers@rivm.nl (M.J.B.M.); 2 Egas Moniz Center for Interdisciplinary Research (CiiEM), Egas Moniz School of Health & Science, Caparica, 2829-511 Almada, Portugal; rassuncao@egasmoniz.edu.pt

**Keywords:** deoxynivalenol, deoxynivalenol-15-glucuronide, deoxynivalenol-3-glucuronide mycotoxin, human biomonitoring, generic PBK modelling, dietary exposure, renal excretion

## Abstract

Toxicokinetic modelling provides a powerful tool in relating internal human exposure (i.e., assessed through urinary biomarker levels) to external exposure. Chemical specific toxicokinetic models are available; however, this specificity prevents their application to similar contaminants or to other routes of exposure. For this reason, we investigated whether a generic physiological-based kinetic (PBK) model might be a suitable alternative for a biokinetic model of deoxynivalenol (DON). IndusChemFate (ICF) was selected as a generic PBK model, which could be fit for purpose. Being suited for simulating multiple routes of exposure, ICF has particularly been used to relate the inhalation and dermal exposure of industrial chemicals to their urinary excretion. For the first time, the ICF model was adapted as a generic model for the human biomonitoring of mycotoxins, thereby extending its applicability domain. For this purpose, chemical-specific data for DON and its metabolites were collected directly from the literature (distribution and metabolism) or indirectly (absorption and excretion) by fitting the ICF model to previously described urinary excretion data. The obtained results indicate that this generic model can be used to model the urinary excretion of DON and its glucuronidated metabolites following dietary exposure to DON. Additionally, the present study establishes the basis for further development of the model to include an inhalation exposure route alongside the oral exposure route.

## 1. Introduction

Mycotoxins are substances produced by fungi that can contaminate plants and plant-derived products during production and storage. Consequently, the general population is predominantly exposed to mycotoxins orally via the intake of food. In addition, occupational exposure can occur alongside oral exposure in certain individuals. The main route of exposure to mycotoxins in an occupational setting is via inhalation as mycotoxins can be present in organic dust. For instance, exposure can occur during the development of working routines in different types of industries (e.g., brewing and bakeries) or during the interaction with feed [1,2,3,4,5,6].

Deoxynivalenol (DON) is usually considered one of the most prevalent mycotoxins, presenting possible adverse health effects for humans and animals. Following acute exposure to DON, acute effects on the gastrointestinal tract of humans and animals have been observed, including vomiting [7]. Chronic exposure has been associated with several adverse health effects in animals, including immune and developmental effects and reduced body weight gain [7,8]. The European Food Safety Authority (EFSA) has derived a group tolerable daily intake (TDI) for DON, DON-3-glucoside (DON-3-G), 3-acetyl-DON (3-A-DON) and 15-acetyl-DON (15-A-DON) of 1 µg/kg body weight/day based on reduced body weight gain in mice [7]. In 2017, EFSA reported that adults sometimes exceeded the group TDI for DON at the 95th percentile of the exposure distribution, while infants, toddlers and children already exceeded the group TDI for DON at the 50th percentile of the exposure distribution [7]. In this scientific opinion, EFSA concluded that although chronic exposure to DON is common worldwide, data related to adverse health effects in humans due to chronic exposure are lacking. To properly assess the associated risks of DON exposure, more robust data should be produced, such as toxicokinetic data. Modelling approaches present a valuable tool to obtain such data.

Traditionally, oral exposure and exposure via inhalation are quantified by means of a dietary survey and by monitoring inhaled particle mass, respectively [9,10]. Alternatively, human biomonitoring can be used to quantify such exposures simultaneously [11]. However, it is necessary to develop a well-defined human kinetic model incorporating both routes of exposure.

Recently, a specific biokinetic model was developed connecting the basic metabolism of DON to its main metabolites, DON-15-glucuronide (DON-15-GlcA) and DON-3-glucuronide (DON-3-GlcA), and subsequent urinary excretion was predicted following oral exposure. This model was based on data from a human intervention study [12]. The mycotoxin model developed by Mengelers et al. (2019) [12] effectively describes the oral exposure of DON and its metabolites for the general population, also when compared to parameters obtained from a real-life situation [13]. However, it is unsuited for the incorporation of the inhalation route of exposure (e.g., contaminated flour dust). In this context, generic physiologically based kinetic (PBK) modelling may be better suited to simulate multi-route mycotoxin exposure.

Generic models can include more routes of exposure and by definition are not limited to simulating the kinetics of only a specific class of chemicals. Therefore, we developed a methodology to define a generic PBK model on the basis of the publicly available biokinetic model by Mengelers et al. (2019) [12] and relevant publicly available data from the literature. Notably, the human urinary biomonitoring data underlying the biokinetic model described by Mengelers et al. (2019) [12] are not publicly available. This PBK model may serve to simulate oral exposure to DON and the corresponding urinary excretion of DON and its metabolites, DON-15-GlcA and DON-3-GlcA, in the general population. Secondly, it may serve as the starting point for further model refinement, such as the incorporation of inhalation exposure to mycotoxins via flour dust particles alongside oral exposure in an occupational setting.

The generic PBK model IndusChemFate (ICF) was selected as the starting point, since it has been previously used for biomonitoring purposes. It also complies with the World Health Organisation (WHO) and the Organisation for Economic Cooperation and Development (OECD) guidelines and it is able to model metabolites next to the parent compound [14,15]. Jongeneelen et al. (2011, 2012) [14,16] demonstrated that the model can be regarded as a screening tool or as a first-tier assessment for industrial chemicals after oral, dermal and inhalation exposure. Fragki et al. (2017) [17] demonstrated that the ICF model was capable of describing the in vivo kinetics of three classes of developmental toxicants, although at the expense of several chemical-specific adaptations. In addition, Pletz et al. (2020) [15] evaluated the use of several generic PBK models/platforms, including ICF, for deriving safe levels in blood or urine (so-called Biomonitoring Equivalents). The authors concluded that ICF and another generic model, High Throughput Toxicokinetics (HttK), were the preferred models because they comply with the WHO and OECD criteria [18,19] for using PBK models in regulatory risk assessment. In short, the OECD published a detailed guidance document with a six-step workflow for characterising and validating PBK models using in vitro and in silico data. This OECD guidance document provides an assessment framework for evaluating PBK models for both PBK model developers and end-users applying the models for regulatory purposes with a focus on uncertainties that underlie the model inputs and outputs [18,20]. Both HttK and ICF concur with these criteria in terms of the description of the model and availability of the model code for checking equations, mass balance and/or making adaptations of the model possible. However, HttK does not include the option to simulate the distribution of the metabolites of the parent compound, whereas ICF includes this option. In addition, ICF is user-friendly and free to download.

The PBK model ICF was first developed to predict concentrations of chemicals and metabolites in alveolar air, blood and urine following environmental or occupational exposure in several populations (including males/females and children) via different routes of exposure (oral, dermal or inhalation) [14,16]). The ICF model can be applied for different exposure durations depending on the exposure route (single bolus versus repeated chronic exposure). The model contains algorithms such as Quantitative Structure–Activity Relationships (QSAR) for calculating blood:air partition coefficients, tissue:blood coefficients and the renal excretion fraction (glomerular filtration and tubular reabsorption). Besides physicochemical input, such as log (octanol:water) partition coefficients (log (Kow)) and water solubility, the model also requires an oral absorption rate constant, resorption fraction in renal tubuli, enterohepatic removal and parameters relevant for metabolism (Km and Vmax) as input. The ICF model does not take renal secretion into consideration.

Here, we adapted the ICF model as a generic modelling tool for the human biomonitoring of mycotoxins, thereby extending its applicability domain. For this purpose, chemical-specific data for DON and its metabolites were collected directly from the literature in order to calculate metabolism rates and partition coefficients. Chemical-specific data were also collected indirectly (absorption and excretion) by fitting the ICF model to the urinary excretion data generated by the oral biokinetic model from Mengelers et al. (2019) [1]. The resulting generic PBK model can be used as a tool in risk assessments relating to internal human exposure (i.e., assessed through urinary biomarker levels) to external dietary exposure. In the future, this generic PBK model can be developed further by the addition of an inhalation route, making it more suitable for an occupational population.

## 2. Results

**Figure 1:**
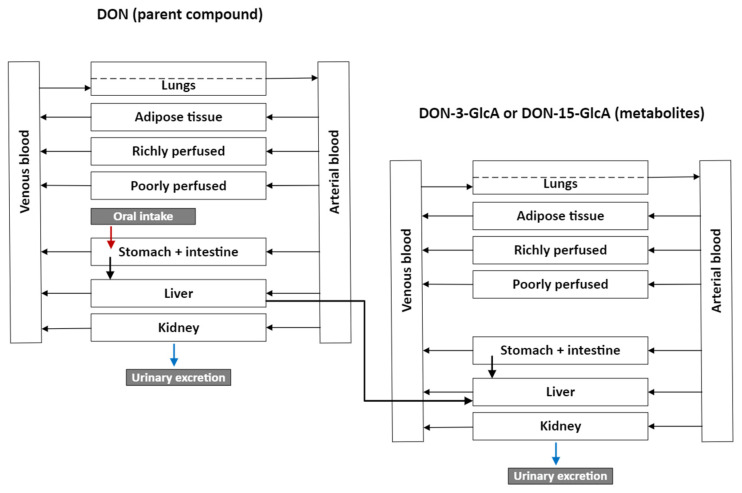
Schematic overview of the DON PBK model containing the compartments liver, kidney, heart, lungs, adipose and stomach and intestine similar to the ICF model and the lumped compartments in the poorly perfused (bone, muscle and skin) and the richly perfused organs (brain, bone marrow).

**Figure 2:**
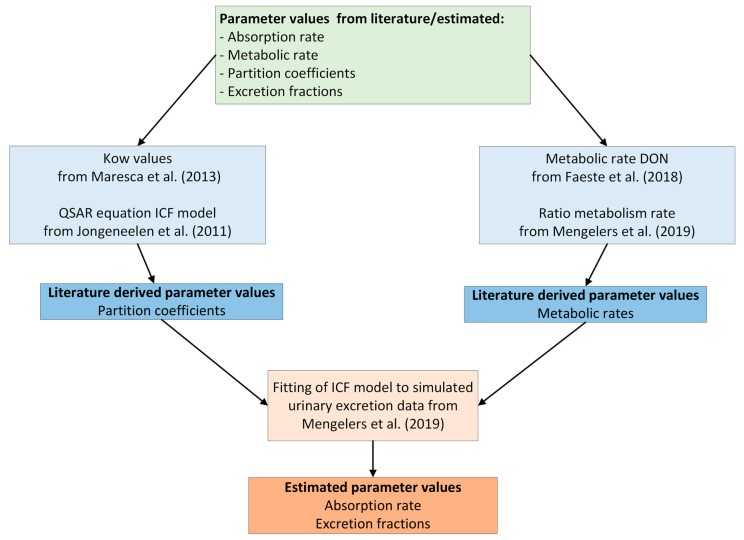
Schematic overview of parameter values for DON and its metabolites that were collected directly from the literature (partition coefficients and metabolism rates) or indirectly (absorption rate constant and excretion fraction) by fitting the ICF model to the urinary excretion data as simulated by the specific biokinetic model developed by Mengelers et al. (2019) [12,14,21,22,23].

**Figure 3:**
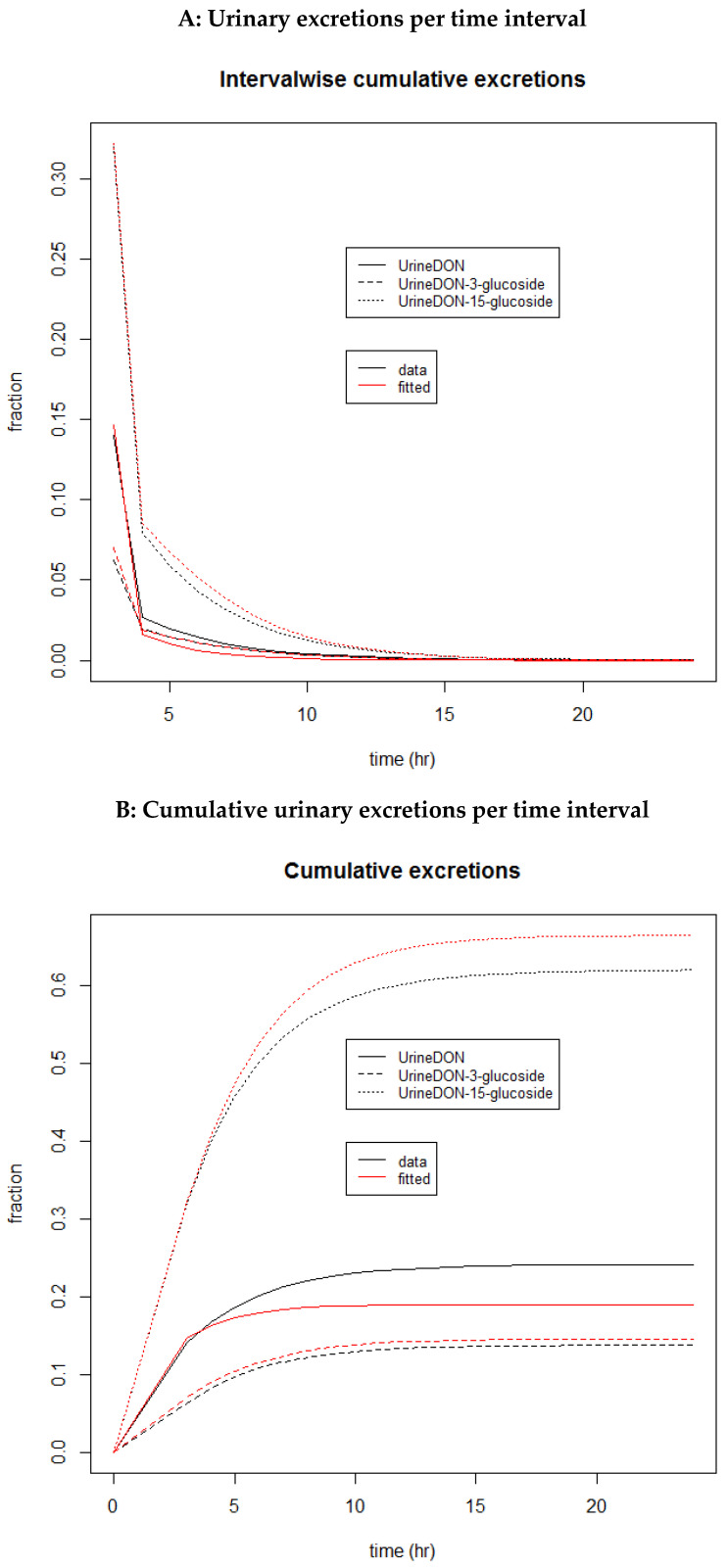
(**A**) Interval-wise renally excreted amounts since the previous reporting time are shown and (**B**) cumulative renally excreted amounts over time, from the starting time point t = 0 for DON and its metabolites, DON-3-GlcA (hyphened line) and DON-15-GlcA (dotted line), based on simulated data from Mengelers et al. (2019) [12] (black lines) and after the fitting of the model (red lines).

**Figure 4:**
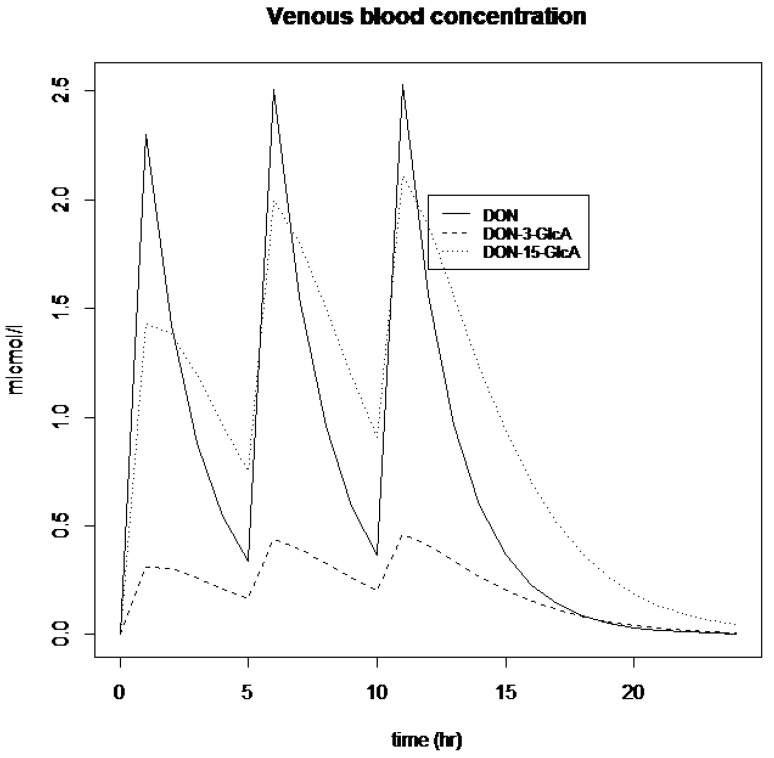
Concentration of DON (line) and its metabolites, DON-3-GlcA (hyphened line) and DON-15-GlcA (dotted line), in blood after a bolus dose of 1 mg DON/kg body weight at t = 0 h, t = 5 h and t = 10 h.

Firstly, the structure of the DON PBK model is described based on the ICF model (Figure 1, Table 1), followed by the calculated parameters based on the publicly available literature (Figure 2, Table 2 and Table 3). Lastly, the absorption rate and renal excretion rate required as input for the adapted ICF model were fitted to the urinary excretion data generated by the oral biokinetic model described by Mengelers et al. (2019) [1] (Table 4). In Figure 3, the resulting DON PBK model (red lines) was compared with the oral kinetic model [1] (black lines). Figure 4 illustrates the DON PBK model.

### 2.1. The DON PBK Model

Figure 1 presents the PBK model applied to DON (parent compound) and its glucuronidated metabolites. The adapted PBK model contains compartments for the liver, kidneys, heart, lungs, adipose tissue, the gastrointestinal tract from which absorption takes place (as depicted by the stomach + intestine) and lumped compartments richly and poorly perfused organs. The model shows that DON is excreted by urine and that faecal excretion is neglectable following absorption. The mathematical equations used in the DON PBK model are similar to that for ICF and can be found in the ICF manual [21]. The adaption of the lumped compartments can be found in the Section 5.1 and the model code can be found in the Supplementary Materials. The calculated allometric data values for the lumped compartments poorly and richly perfused tissues can be found in Table 1.

**Table 1:** Allometric data values for the poorly and richly perfused tissues compartments.

Parameters	Poorly Perfused Organs	Richly Perfused Organs
Relative volume	0.58	0.09
Relative flow	0.35	0.18

### 2.2. Calculation and Estimation of DON-Related Parameters

To adapt the ICF model as a generic modelling tool for the human biomonitoring of mycotoxins, relevant DON-related parameters were retrieved from the existing literature (i.e., organ/blood partition coefficients and metabolism-related parameters) or estimated (i.e., absorption rate constant, and excretion-related parameters through fitting the adapted ICF model to excretion amounts that were generated by the biokinetic model from Mengelers et al. (2019) [12] (see Section 5.2 and Figure 2).

#### 2.2.1. Calculation of DON-Related Parameters Based on Data from the Literature

The DON-related parameters that were calculated and/or retrieved from the literature were the organ/blood partition coefficients and the metabolism-related parameters. The (organ-specific) partition coefficients were calculated using QSARs, based on physicochemical data of DON and its metabolites (Table 2).

**Table 2:** Physicochemical input parameter values for DON and its main metabolites used in the model (retrieved from Maresca et al. (2013) [22]).

Parameters	DON	DON-3-GlcA	DON-15-GlcA
Log (Kow)	−9.7	−5.75	−5.75
Water solubility	5.5 × 10^4^	5.5 × 10^4^	5.5 × 10^4^

Table 3 summarises the resulting calculated organ-specific partition coefficients for DON and its metabolites, based on the QSAR calculations used in the ICF model and the parameter values are presented in Table 2.

**Table 3:** Calculated organ-specific partition coefficients ^1^ for DON and its metabolites.

Compartment	DON	DON-3-GlcA	DON-15-GlcA
Adipose tissue	0.1	0.1	0.1
Poorly perfused	0.74	0.74	0.74
Richly perfused	0.78	0.78	0.78
Kidney	0.77	0.77	0.77
Intestine	0.52	0.52	0.52
Liver	0.52	0.52	0.52
Lung	0.74	0.74	0.74

^1^ Note: In ICF, at low log (Kow), organ:blood partition coefficients are determined by the water/lipid content of the blood and the organs. Hence, all calculated partition coefficients are identical for all forms of DON. Furthermore, in ICF, the adipose tissue:blood ratio has a minimum value of 0.1.

In the ICF model, metabolism is described using the parameters Vmax (maximum velocity of metabolism) and Km (Michaelis–Menten constant). These parameters were calculated as follows. The metabolic rate retrieved from the literature was the intrinsic clearance of DON of 0.39 L/hour × kg body weight [23]. In combination with the ratio of the two metabolisation rate values from Mengelers et al. (2019) [12] for the parallel generation of DON-3-GlcA and DON-15-GlcA, a metabolic rate of 0.07 and 0.32 L/hour × kg body weight, respectively, was calculated. The Vmax parameter value was estimated (as described in the Section 5.2.1. and Supplementary S1), given the large value of the Km parameter for each substance. In the last calculation step, it was assumed that the metabolism process in our model was almost linear with the ratio Vmax/Km being the metabolic rate value, in accordance with the model described in Mengelers et al. (2019) [12].

#### 2.2.2. Estimation of DON-Related Parameters by Fitting

Excretion in the urine is modelled in the ICF model as a linear combination of the Glomerular Filtration Rate, the fraction of the substance in arterial blood that is dissolved in water and the fraction of the dissolved substance that is excreted in the urine, the latter depending on the Kow. However, fitting the DON PBK model with ICF’s default Kow dependent renal excretion fraction value did not result in a good fit. Therefore, it was necessary to re-calibrate the renal excretion fraction. As a result, two parameters still needed to be estimated, the absorption rate constant and the renal excretion fraction.

The absorption rate constant and the renal excretion fraction were calculated by fitting the PBK model to the results of the biokinetic model developed by Mengelers et al. (2019) [12]. Their values are shown in Table 4. It should be noted that, since we started with a bolus intake of value 1, and the model is completely linear, the excretion amounts can be interpreted as fractions. Remarkably, the excretion fraction of the glucuronides is above 1, indicating a renal secretion step.

**Table 4:** Estimated parameter values after fitting the PBK model to the results of the biokinetic model from Mengelers et al. (2019) [12].

Parameter	DON	DON-3GlcA	DON-15GlcA
Absorption rate constant	22 (per hour)		
Renal excretion fraction	0.97	3.28	3.28

In Figure 3A, the excreted amounts per 1 h time interval are shown after a single oral dose, starting with the first excretion measurement after 3 h. This means that the presented values show the excreted amounts at voiding time points with 1 h time intervals, after an initial 3 h time interval. In addition, in Figure 3B, the cumulative excreted amounts over time for all absorption time points, i.e., the cumulative excretion from the starting time point t = 0, are shown. Note that the model was fitted on the interval-wise excretion amount values as depicted in Figure 3A. The latter results show that all interval-wise excreted amounts calculated this way resulted in overall cumulative excretion amounts for DON-3-GlcA and DON-15-GlcA that differed only slightly from the values that resulted from the biokinetic model from Mengelers et al. (2019) [12]. Moreover, as seen in Figure 3B, the fitting procedure resulted in good fits for the lower reporting time points, where most of the DON intake was excreted as expected.

### 2.3. Illustration of the DON PBK Model

The DON PBK model can be used to develop a human biomonitoring sampling strategy, resulting from real-life exposure situations. To illustrate this, we assumed three oral exposures during the day (e.g., during breakfast, lunch and dinner). Each of these exposures was modelled as a bolus of 1 mg DON. Figure 4 shows the blood concentration of DON and its metabolites following these real-life exposure scenarios. As expected, the blood concentrations of DON and its metabolites showed rapid changes during the day following oral intake, indicating that monitoring this time course requires an accurate sampling strategy.

## 3. Discussion

Similar to the original ICF model, the PBK model that we developed for DON does not include a fractional absorption parameter (fabs). That means that the model assumes that 100% of the intake also enters the liver compartment, and thus the human body. Previous studies [12,13] have shown that only a fraction (i.e., 0.44–0.56) of DON is absorbed by the human body and that this fraction is highly heterogeneous in human populations. Therefore, to make the model useful for human biomonitoring applications, a stochastic fractional absorption parameter should be included in future modelling exercises.

When comparing different PBK models, several similarities and dissimilarities become apparent. For example, several organs are consistently included in these models because of their specific functionality, e.g., kidney for excretion, liver for metabolism, gastrointestinal tract for absorption following oral intake, lungs for inhalation and exhalation (when applicable), and skin for dermal absorption (when applicable). PBK models differ with respect to those organs (compartments) that are relevant for internal storage or for the specific organ toxicity of the substance in the human body, e.g., brain and muscles. For these reasons, the ICF model and the Httk PBK model include a much larger set of specific organs compared to other models. However, since the in-flow and out-flow of substances in the ICF model are modelled as a linear process (i.e., proportional to the blood flow), compartments can easily be combined. The parameters to be changed were the compartment volumes and blood flows, and the partition coefficients. In cases of aggregating organs into one compartment, volumes and flow values must be added, and the partition coefficient of the new compartment equals the weighted sum of the partition coefficients of the organs.

In our study, unknown model parameters were estimated either by calculating them directly from the data retrieved from the literature, e.g., the partition coefficients and the metabolism rates, or by calculating them indirectly by fitting the adjusted ICF model, the DON PBK model, to the biokinetic model by Mengelers et al. (2019) [12] using the calculated excretion amounts (model calibration). The advantage of direct estimation is the one to one relation between the data and parameter(s), whereas a disadvantage may be the different contexts; the data value may come from an animal experiment or an in vitro environment, whereas the application involves a human in vivo situation. The model calibration process guarantees that the environments of both models are the same. Since the biokinetic model from Mengelers et al. (2019) [12] is fitted to the collected human biomonitoring data, both the biokinetic and PBK-model describe these data. The disadvantage of model calibration is the issue of parameter identifiability. It is not automatically guaranteed that the parameter values calculated by model calibration are realistic. This issue occurs when the parameters are highly correlated. In that case, rather different combinations of parameter values may lead to very similar values of the model output variables. In mathematics, this is known as the model identifiability problem. In our analyses, the problem occurred when we tried to simultaneously calculate the absorption and metabolic rates, and the excretion fractions through model calibration. The procedure was unsuccessful due to the correlations between these parameters. Unsuccessful means that we were mathematically not able to assess clear parameter values, only relatively large intervals of parameter values that all resulted in similar model fits. For this reason, we were forced to calculate one of the three unknown parameters directly from the data retrieved from the literature, allowing the other two parameters to be identified by model calibration. In our case, the metabolic rate was calculated by combining in vitro data from Faeste et al. (2018) [23] and parameter values from the biokinetic model from Mengelers et al. (2019) [12].

Two important aspects can be distinguished with respect to the parameters of the PBK-model: heterogeneity and uncertainty. Heterogeneity means that parameter values can be different over time within individuals, and also between individuals. These forms of variability are known as within-variability and between-variability, respectively. Pletz et al. (2020) [15] showed that the latter form is more important. Previously, it was shown that the between-variability of the fraction absorbed is large in human populations for DON [12,13]. Regarding uncertainty, each parameter’s value is uncertain, due to imperfect measurements. Pletz et al. (2020) [15] showed that errors may occur in the calculation of in vivo model parameters from results of in vitro experiments. These errors may consist of the following two terms: firstly, a variance term, indicating imprecision, and secondly, a bias term, indicating a systematic over- or underestimation.

An example of bias is the modelling of the renal excretion fraction, described by the glomerular filtration rate and tubular resorption fraction [14,21]. Our results and those from Pletz et al. (2020) [15] indicate that this way of modelling underestimates the excretion of highly soluble substances such as DON. This resulted in modelling in terms of the presence of active renal secretion in our study. Another example of (possible) bias relates to the validity of the partition coefficients using the QSAR equation applied in the ICF model, which uses the log (Kow) value as an input variable. The log (Kow) values of DON and its metabolites are small, resulting in partition coefficients that are close to 1, which show that DON is highly soluble. This raises the question whether the QSAR equation is applicable for such small log (Kow) values.

As previously mentioned, heterogeneity can be distinguished between within-person changes over time, and between-person differences. The parameter values of the compartmental DON model were partly based on the parameter values of the ICF model [21], partition coefficients and metabolisation rates from the literature. Conditional to these parameter values, the absorption and excretion rates were calculated by calibration of the biokinetic model from Mengelers et al. (2019) [12]. The ICF model deals with heterogeneity by providing allometric parameter values for different types of humans, e.g., normal weight versus obese, or men versus women. However, the partition coefficients and metabolisation rates from the literature and the biokinetic model state-transition rates were only presented as population mean values with 95% confidence bounds. Therefore, as a result, it is possible to describe the population heterogeneity in the DON PBK model in terms of different allometric parameter values, whereas the other parameter values are assumed to be homogenous instead of heterogeneous. In future studies, it is possible to reduce the heterogeneity by looking at sub-populations.

DON belongs to a group of mycotoxins that are called ‘trichothecenes’. Chemically, trichothecenes share a tetracyclic sesquiterpenoid 12,13-epoxy-trichothec-9-ene ring system and are divided into four groups (A–D) according to different functional groups. Unlike types C and D, types A and B are frequently found as contaminants in cereals and other commodities [1]. DON is a type B trichothecene and it would be worthwhile to investigate whether this generic model can also be used to study the kinetic behaviour of type A trichothecenes, like T-2/HT-2 toxin, in humans. For example, there is an important similarity between DON and T-2/HT-2 toxins regarding their elimination in mammals, as both types of trichothecenes are subject to glucuronidation and renal excretion [7,24].

The PBK model we developed based on the ICF model has been fitted for oral exposure. This model is based on the ICF model, which is suitable for several exposure routes [14], including the intake of DON through inhalation. The basis is already there, since the original ICF model already includes absorption via inhalation. However, modelling the inhalation of DON is considerably more complex. Exposure to DON through contaminated flour dust particles was already reported [5,9]. DON flour dust particles are relatively large, and are in essence non-gaseous. Consequently, the ICF’s model assumption of the substance being in a gaseous state where the exposure can be described by a gaseous concentration does not apply to DON. Instead, this exposure should be described in a more complex way. DON molecules could be attached to flour dust particles that differ in size. The mean flour dust concentrations differ between occupations, e.g., dough-makers and oven-workers, which is also a factor to be properly considered [25]. Moreover, the average size of the particles is about 80 microns (PM 80), with the smallest about 10 microns (PM 10) and the largest 400 microns [26,27]. In cases of sizes that are smaller than 80 microns [25], the particles enter the lungs through inhalation, are deposited in the lungs, and enter the human body through alveolar deposition, or trachea–bronchial deposition [27,28]. In cases of larger sizes, the particles cannot enter the lungs, but instead are being coughed up, and then partly enter the human body via gastrointestinal absorption after swallowing. In addition, during risk assessment including this inhalation exposure route, toxicity of the fungi-producing DON should be taken into account next to toxicity caused by DON, since the fungi (spores) themselves can also cause health effects, such as infection, allergy and inflammation [29,30].

The current PBK-model for DON heavily relies on the biokinetic model described by Mengelers et al. (2019) [12], which was built using data from a human intervention study. This results in questions on the range of application of the PBK model. For instance, the absorption may differ in the case of DON intake through day-to-day consumption instead of an experimental dose. Yet, modelling the relation between DON intake and excretion in an everyday situation with multiple consumption moments in Norwegian volunteers revealed similar excretion and time-to-excretion parameters as obtained in the human intervention study [13]. Therefore, further model validation using empirical data is necessary.

## 4. Conclusions

DON is usually considered one of the most prevalent mycotoxins, presenting possible adverse health effects for humans and animals. Consequently, robust data should be produced to properly assess the associated risk of DON exposure, such as toxicokinetic data. Modelling approaches are usually used in this context as valuable tools to obtain such data. In the present study, we have applied, for the first time, a generic PBK model by adapting the ICF model. This way, generated data could be used in the context of human biomonitoring and health risk assessment. Our results showed that for DON and its glucuronidated metabolites, a PBK model was successfully developed and its parameters estimated. Despite the associated identified limitations, this PBK model can be used for the biomonitoring of the dietary DON intake. Recognising that exposure routes other than oral are possible, such as inhalation, constitutes an important aspect, particularly in the context of occupational exposure. The present study establishes the basis for the further development of the model to include an inhalation exposure route alongside an oral exposure route.

## 5. Materials and Methods

A PBK model was developed based on the already existing PBK model, IndusChemFate (ICF). To calibrate the model, literature data and simulated human biomonitoring data, resulting from the biokinetic model described by Mengelers et al. (2019) [12], were used (Figure 2). All models were written in the programming language R. The source code is provided in the Supplementary S2 and the source codes for the model fitting procedures are available upon request.

### 5.1. General Description of the PBK Model

The ICF model [14,21] was used as a starting point. This model simulates the absorption, distribution, metabolism and excretion of chemicals. The main input data are allometric data, e.g., blood flows and organ volumes, and substance-specific data, e.g., partition coefficients and metabolism rates. The multicompartmental ICF model contains compartments for the liver, kidneys, heart, lungs, adipose tissue and a compartment for the stomach and intestine. As shown in Figure 1 (Section 2.1), the ICF model was simplified by dividing the remaining organs in two compartments, the poorly perfused (bone, muscle and skin) and the richly perfused organs (brain and bone marrow). For these combined compartments, the combined organ volumes and flows were calculated as the sum of the separate ICF volumes and flows, respectively. According to the ICF model [14,21], the same QSAR equation was used to calculate the partition coefficients for each of the organs/compartments separately. The resulting PBK model, and its kinetic parameters, for the oral intake of DON by humans are given in the Section 2 (Figure 1, and Table 2 and Table 3).

### 5.2. DON-Related Parameters

The unknown DON-related parameters to be calculated were the organ/blood partition coefficients, the absorption rate constant, the metabolism-related parameters, and the excretion-related parameters. In Figure 2 (Section 2.2), a schematic overview is given of the parameter values that were directly derived from the literature (partition coefficients and metabolism rates) or indirectly (absorption rate constant and excretion fraction) by fitting the ICF model to the urinary excretion data as simulated by the specific biokinetic model described by Mengelers et al. (2019) [1].

#### 5.2.1. Deriving DON-Related Parameters from the Literature

The partition coefficients were calculated from the Know value for DON reported in the literature [22] using the QSAR equations mentioned in Section 5.1 above.

Metabolism was described in the ICF model as a restricted process including two parameters, the Vmax parameter (the maximum velocity of metabolism) and Km (the Michaelis–Menten constant). However, in the biokinetic model [12], the metabolism of DON was modelled as a linear process, meaning that the rate of metabolism of DON was assumed to be proportional to the amount of DON without any restriction. As a result, we could only calculate the ratio Vmax/Km from the data available, which can be interpreted as the metabolism rate constant value. To calculate the total metabolism rate constant value (i.e., the sum of the metabolism rate constant values for DON-3-GlcA and DON-15-GlcA), we used data from Faeste et al. (2018) [23]. In this article, the metabolic rate value of DON was presented as 0.39 L/hour × kg body weight. The ratio of the metabolism rate constant values for DON-3-GlcA and DON-15-GlcA could be assessed from Mengelers et al. (2019) [12]. For Km, we selected a value much larger than the DON concentrations that resulted from the data intake values. For the specific mathematical calculation steps, see Supplementary S1. Note, as a consequence, the resulting PBK model formally only applies to situations where the intake of DON is not much larger than the intake values applied by Mengelers et al. (2019) (i.e., 1 µg/kg body weight).

#### 5.2.2. Deriving DON-Related Parameters by Fitting of ICF Model: Calculation of the PBK-Model Absorption and Renal Excretion Parameters

The set of unknown parameters included the bolus absorption rate constant and two renal excretion fractions for both metabolites. The excretion-related parameter to be estimated is the parameter named RemovKidney in the ICF model (i.e., the fraction excreted in urine). This is the fraction removed from the glomerulus filtrate minus the fraction resorbed by the renal tubuli and excreted with urine. Similar to the biokinetic model, we assumed the excretion fractions of DON-3-GlcA and DON-15GlcA to be equal to that for DON. These parameters were estimated by fitting the PBK model to the excretion amounts that were generated by the biokinetic model from Mengelers et al. (2019) [12]. Therefore, given one bolus intake of DON at time point 0 (hour), the biokinetic model from Mengelers et al. (2019) [12] was used to calculate cumulative excretion amounts for each substance for all successive 1 h time intervals. Then, the PBK model was fitted to these excretion amounts with the bolus absorption rate constant and the two renal excretion fractions as the unknown parameters. The criterium function to be minimised in the fitting procedure was the squared differences between the data (biokinetic model) and the calculated (PBK model) excretion amounts, being aggregated over all time intervals and over all substances (method of least sum-of-squares). Since in the work of Mengelers et al. (2019) [12], the first excretions were measured approximately 3 h after intake, we mimicked this in our procedure by starting with a 3 h excretion amount, followed by 1 h excretion amounts.

## Data Availability

Not applicable.

## Supplementary Materials

The following supporting information can be downloaded at: https://www.mdpi.com/article/10.3390/toxins15090569/s1, S1: Calculation of Vmax and Km, S2: R-code of DON PBK model.
