# Supplementary material for: Development of a Generic PBK Model for Human Biomonitoring with an Application to Deoxynivalenol
Source: Toxins (Basel). 2023 Sep 13;15(9):569. doi: 10.3390/toxins15090569 (PMC10535232; doi:10.3390/toxins15090569)
Supplement: Supplementary file 1 [file toxins-15-00569-s001.zip › toxins-2454448-supplementary.pdf]

Supplementary S1

The calculation of the two metabolism parameters Vmax (maximum velocity of metabolism) and Km (Michaelis– Menten constant), that are input to the ICF-model, was done as follows: The intrinsic clearance reported by Faeste et al. (2018) [23] is 0.39 [L/h x kg bodyweight]. In the PBK model two parallel metabolization processes take place, generation of DON-3-GlcA and DON-15-GlcA respectively. The biokinetic model from Mengelers et al. (2019) [12] gives information on the ratio of the two metabolization rate values: the ratio of the latter to the former rate value is 3.29 / 0.73. The sum is 0.39 [L/h x kg bodyweight], so, the two metabolization rates are 0.07 and 0.32 [L/h x kg] respectively. The biokinetic model describes a linear metabolization processes, without any practical restriction. For large values of Km the intrinsic clearances are approximately equal to the ratio of Vmax to Km. Thus, since we cannot independently estimate the Vmax and the Km value of the metabolization processes, we assumed equal, large Km-values (1000) for both metabolization processes, and calculated the Vmax-values that result in the metabolization rates given above:

DON-3-GlcA: Vmax = .07 * BW * Km = .07 * 70 * 1000 = 4900 L/h

DON-15-GlcA: Vmax = .32 * BW * Km = .32 * 70 * 1000 = 22400 L/h

Note, the absolute values of Vmax and Km have no empirical foundation here, only the ratio.

Supplementary 2

The part of the code that describes the right-hand side of the differential equations is given below. It describes the change of the amounts in all compartments given the current amounts for any time point and the model parameter values.

```
#--------------------------------------------------------------------------------

# version of model in terms of differential equations that can be solved using package deSolve

# ode-routine works with a vector of state variables that is updated; within the routine that calculates

# the gradient the vector is split up in two arrays, one on the amounts of the substances in the organs,

# one on the other amounts in lung etc.

# wherever meaningful, parameter extensions are used , e.g. arrays of volumes of compartments for all

# substances

#--------------------------------------------------------------------------------

# Organs: (1) Adipose tissue, ..

# Other amounts: (1) Arterial blood entering organs, (2) Venous blood entering lung ..


f.program2 <- function(input, parlist) {


NumSubs      <- input$NumSubs
extind       <- input$extind
serialmetab  <- input$serialmetab


FlowOrg      <- parlist$FlowOrg
Organ        <- parlist$Organ
NumOrgan     <- length(Organ)
Vmax         <- parlist$Vmax
VolCmp       <- parlist$VolCmp
Vext         <- parlist$Vext
extind1      <- parlist$extind1
extind2      <- parlist$extind2
extind3      <- parlist$extind3
extind01     <- parlist$extind01
RCtisbl      <- parlist$RCtisbl
PA           <- parlist$PA
timind       <- parlist$timind
timval       <- parlist$timval
ntimval      <- length(timval)
timrep       <- parlist$timrep
ntimrep      <- length(timrep)
Chem         <- parlist$Chem
MgBolus      <- parlist$MgBolus
BodyWt       <- parlist$BodyWt
MW           <- parlist$MW
```

```
CardOutp     <- parlist$CardOutp
Iintest      <- parlist$Iintest
Iliver       <- parlist$Iliver
Ikidney      <- parlist$Ikidney
Iadip        <- parlist$Iadip
Ipoorperf    <- parlist$Ipoorperf
Irichperf    <- parlist$Irichperf
VolBlungArt  <- parlist$VolBlungArt
VolBlungVen  <- parlist$VolBlungVen
AlvVent      <- parlist$AlvVent
RCba         <- parlist$RCba
RemovKdn     <- parlist$RemovKdn
Kmime        <- parlist$Kmime
EntHepRt     <- parlist$EntHepRt
DecrBolusRt  <- parlist$DecrBolusRt
GlomFiltr    <- parlist$GlomFiltr
FrWsol       <- parlist$FrWsol
FaecesFract  <- parlist$FaecesFract
Cexp         <- parlist$Cexp
RespProt     <- parlist$RespProt

amrestnames <- c("Artblood", "Venblood", "lungArt", "lungVen", "Lumen", "Bolus", "Urine",
"Faeces", "Metab",
                 "Exhale", "Inhale")
namrest      <- length(amrestnames)
Iartblood    <- (1:namrest)[amrestnames == "Artblood"]
Ivenblood    <- (1:namrest)[amrestnames == "Venblood"]
IlungArt     <- (1:namrest)[amrestnames == "lungArt"]
IlungVen     <- (1:namrest)[amrestnames == "lungVen"]
Ilumen       <- (1:namrest)[amrestnames == "Lumen"]
Ibolus       <- (1:namrest)[amrestnames == "Bolus"]
Iurine       <- (1:namrest)[amrestnames == "Urine"]
Ifaeces      <- (1:namrest)[amrestnames == "Faeces"]
Imetab       <- (1:namrest)[amrestnames == "Metab"]
Iexhale      <- (1:namrest)[amrestnames == "Exhale"]
Iinhale      <- (1:namrest)[amrestnames == "Inhale"]
Subcompnames <- c("extracell", "intracell")

# state variables: organs (total or extra/intra cellular parts),
# AmtlungArt/Cblart, AmtlungVen/CBlungVen, lumen, bolus

parDElist    <- list(amrestnames = amrestnames, IlungVen = IlungVen,
                 IlungArt = IlungArt, Ibolus = Ibolus, Iurine = Iurine,
                 Ilumen = Ilumen, Ifaeces = Ifaeces, Imetab = Imetab,
                 Iexhale = Iexhale, Iinhale = Iinhale)
```

```
#--------------------------------------------------------------------------------
# parameter values extended to state variable formats
#--------------------------------------------------------------------------------

FlowOrgj1      <- array(FlowOrg, dim = c(NumOrgan, NumSubs))
dimnames(FlowOrgj1) <- dimnames(Vmax)

VolCmpi1       <- array(VolCmp, dim = c(NumOrgan, NumSubs))
dimnames(VolCmpi1) <- dimnames(Vmax)

#--------------------------------------------------------------------------------
# calculates gradient
#--------------------------------------------------------------------------------

f.diff1 <- function(ht, am, parms = 0) {

    VolCmpi    <- VolCmpi1
    CardOutpi <- CardOutp
    FlowOrgi   <- FlowOrgj1

    #--------------------------------------------------------------------------------
    # cardiac output equal to sum of organ flows
    #--------------------------------------------------------------------------------

    CardOutpi                <- sum(FlowOrgi[ , 1])

    #--------------------------------------------------------------------------------
    # amounts in organs
    #--------------------------------------------------------------------------------

    Amj1                                   <- array(am[1:(NumOrgan * NumSubs)], dim = c(NumOrgan,
NumSubs))
    dimnames(Amj1)       <- dimnames(Vmax)

    #--------------------------------------------------------------------------------
    # amounts in arterial blood, venous blood, intestine lumen, bolus
    #--------------------------------------------------------------------------------

    Amjrest                  <- array(am[-(1:(NumOrgan * NumSubs))], dim = c(namrest, NumSubs))
    dimnames(Amjrest)   <- list("Organ" = amrestnames, dimnames(Vmax)[[2]])

    dAmjrest                 <- 0 * Amjrest

    #--------------------------------------------------------------------------------
    # concentration in organs
    #--------------------------------------------------------------------------------
```

```
Concorg    <- VolCmpi * Amj1 / (VolCmpi^2 + eps)


#--------------------------------------------------------------------------------
# concentration in organs (blood-adjusted)
#--------------------------------------------------------------------------------

Cblorg       <- Concorg / RCtisbl


#--------------------------------------------------------------------------------
# concentration in arterial blood available for organs resp. defined for all organs
#--------------------------------------------------------------------------------

Cblart       <- Amjrest[IlungArt, ] / VolBlungArt

Cblarti      <- t(array(Cblart, dim = c(NumSubs, NumOrgan)))


#--------------------------------------------------------------------------------
# concentration in venous blood entering lung
#--------------------------------------------------------------------------------

CBlungVen                <- Amjrest[IlungVen, ] / VolBlungVen


#--------------------------------------------------------------------------------
# concentration in arterial blood leaving lung
#--------------------------------------------------------------------------------

CBlungArt <-  (CardOutpi  *  CBlungVen  +  AlvVent  *  Cinh)  /  (CardOutpi  +  AlvVent  /  RCba)

dAmjrest[Iinhale, ] <- AlvVent * Cinh
dAmjrest[Iexhale, ] <- AlvVent * CBlungArt / RCba


#--------------------------------------------------------------------------------
# change of amount in arterial blood leaving lung
#--------------------------------------------------------------------------------

dAmjrest[IlungArt, ] <- CardOutpi * (CBlungArt - Cblart)


#--------------------------------------------------------------------------------
# amount and concentration in venous blood having left organs
#--------------------------------------------------------------------------------

Amorgven <- apply(array(FlowOrgi[-c(Iintest, Iliver), ] * Cblorg[-c(Iintest, Iliver), ],
                                                  dim = c(NumOrgan - 2, NumSubs)), 2, sum) +
```

```
                              apply(array(FlowOrgi[c(Iintest, Iliver), ], dim = c(2,
NumSubs)), 2, sum) * Cblorg[Iliver, ]
    Cblven      <- Amorgven / CardOutpi



    #--------------------------------------------------------------------------------
    # amount in venous blood volume
    #--------------------------------------------------------------------------------

    dAmjrest[IlungVen, ] <- CardOutpi * (Cblven - CBlungVen)



    #--------------------------------------------------------------------------------
    # amount biotransformed out of liver resp. into liver
    #--------------------------------------------------------------------------------

    QntDecr    <- rep(0, NumSubs)
    QntIncr    <- rep(0, NumSubs + 1)
    if (serialmetab) {
            QntDecr <- (Kmime > 0) * Vmax * VolCmpi[Iliver, ] * Concorg[Iliver, ] / (Kmime +
Concorg[Iliver, ] + eps)
            QntIncr[-1] <- (Kmime > 0) * Vmax * VolCmpi[Iliver, ] * Concorg[Iliver, ] /
                    (Kmime + Concorg[Iliver, ] + eps)
    } else {
            QntDecr[1] <- sum((Kmime[-NumSubs] > 0) * Vmax[-NumSubs] * VolCmpi[Iliver, 1]
            * Concorg[Iliver, 1] /
                    (Kmime[-NumSubs] + Concorg[Iliver, 1] + eps))
            QntIncr[2:NumSubs] <- (Kmime[-NumSubs] > 0) * Vmax[-NumSubs] *
VolCmpi[Iliver, 1] *
                    Concorg[Iliver, 1] / (Kmime[-NumSubs] + Concorg[Iliver, 1] + eps)
    }

    #--------------------------------------------------------------------------------
    # generic mass flow into/out of organs
    #--------------------------------------------------------------------------------

    dAmj1             <- FlowOrgi * (Cblarti - Cblorg)



    #--------------------------------------------------------------------------------
    # extra mass flow into/out of liver
    #--------------------------------------------------------------------------------

    dAmj1[Iliver, ] <- dAmj1[Iliver, ] + FlowOrgi[Iintest, ] * (Cblorg[Iintest, ] - Cblorg[Iliver, ]) -
                        EntHepRt * Amj1[Iliver, ] + QntIncr[1:NumSubs] - QntDecr
    dAmjrest[Imetab, ] <- - QntIncr[1:NumSubs] + QntDecr


    #--------------------------------------------------------------------------------
```

```
    # extra mass flow into/out of intestine
    #--------------------------------------------------------------------------------

    dAmj1[Iintest, ] <- dAmj1[Iintest, ] + .3 * Amjrest[Ilumen, ] + DecrBolusRt * Amjrest[Ibolus, ]

    #--------------------------------------------------------------------------------
    # extra mass flow out of kidney
    #--------------------------------------------------------------------------------

    dAmj1[Ikidney, ] <- dAmj1[Ikidney, ] - GlomFiltr * RemovKdn * FlowOrgi[Ikidney, ] * Cblart *
FrWsol
    dAmjrest[Iurine, ] <- dAmjrest[Iurine, ] + GlomFiltr * RemovKdn * FlowOrgi[Ikidney, ] * Cblart
* FrWsol

    #--------------------------------------------------------------------------------
    # mass flow into/out of intestinal lumen and into faeces
    #--------------------------------------------------------------------------------

    dAmjrest[Ilumen, ] <- (1 - FaecesFract) * EntHepRt * Amj1[Iliver, ] - .3 * Amjrest[Ilumen, ]
    dAmjrest[Ifaeces, ] <- FaecesFract * EntHepRt * Amj1[Iliver, ]

    #--------------------------------------------------------------------------------
    # mass flow out of bolus
    #--------------------------------------------------------------------------------

    dAmjrest[Ibolus, ] <- - DecrBolusRt * Amjrest[Ibolus, ]

    return(list(c(c(dAmj1), c(dAmjrest)))) }

#--------------------------------------------------------------------------------
# all calculation steps
#--------------------------------------------------------------------------------

if (extind01[1]) {

    amrep1    <- array(0, dim = c(ntimrep, NumSubs * (NumOrgan + namrest)))
    hnaam              <- c(c(outer(Organ, Chem, paste, sep = "")),
                            c(outer(amrestnames, Chem, paste, sep = "")))
    dimnames(amrep1) <- list("time" = 1:ntimrep, "comp" = hnaam)
    am1                <- rep(0, NumSubs * (NumOrgan + namrest))
    j                  <- 1
    Cinh               <- rep(0, NumSubs)

    for (i in 1:(ntimval - 1)) {
            if (timind[i] == "eenbolus") {
                    am1[NumSubs * NumOrgan + Ibolus] <-
```

```
                                am1[NumSubs * NumOrgan + Ibolus] + 1000 * MgBolus * BodyWt / MW[1]
            }
            if (timval[i] != timval[i + 1]) {
                  if (timind[i] == "rep") { amrep1[j, ] <- am1; j <- j + 1 }
                  ham       <- ode(am1, timval[c(i, i + 1)], f.diff1)
                  am1       <- ham[2, -1]
            }
   }

   #--------------------------------------------------------------------------------
   # function generates time-dependent model output variables based on the reported substance amount values
   #--------------------------------------------------------------------------------

   tableall <- f.writeplotres1(amrep1, "DEnew", input, parlist, parDElist)
}

hres        <- list(amrep = amrep1, tableall = tableall, message = "tableall", parDElist = parDElist)

return(hres) }
```
